# General practitioners versus other physicians in the quality of primary care: a cross-sectional study in Guangdong Province, China

**DOI:** 10.1186/s12875-015-0349-z

**Published:** 2015-10-09

**Authors:** Yaming Zou, Xiao Zhang, Yuantao Hao, Leiyu Shi, Ruwei Hu

**Affiliations:** Department of Medical Statistics and Epidemiology, School of Public Health, Sun Yat-sen University, 74 Zhongshan Road 2, Guangzhou, 510080 P.R China; Department of Health Policy & Management, Bloomberg School of Public Health, Primary Care Policy Center, Johns Hopkins University, Baltimore, USA; Department of Health Management, School of Public Health, Sun Yat-sen University, 74 Zhongshan Road 2, Guangzhou, 510080 P.R China

**Keywords:** General practitioners, Quality of primary care, China, Primary Care Assessment Tool (PCAT)

## Abstract

**Background:**

The primary care in China can be provided by general practitioners (GPs) and other physicians (non-GPs). However, China’s general practice system has never been really established. Chinese patients tend to consider the quality of primary care provided by GPs much lower than that of non-GPs. Besides, many GPs presently prefer leaving their own positions and seeking better development in big hospitals, which has made the already weak GP system weaker. Yet, few studies have specially compared the quality of primary care provided by Chinese GPs and other physicians and no studies have explored the independent predictors of Chinese GPs’ intentions to stay on their current job. In this study, we aimed to compare the quality of primary care offered by GPs with non-GPs and to explore the independent predictors of GPs’ future work intentions.

**Methods:**

This cross-sectional study applied multi-stage random cluster sampling methodology. The data were collected from November 2013 to September 2014 in Guangdong Province. In total, 401 effective questionnaires were selected from the physicians. Quality of primary care was assessed using the Primary Care Assessment Tool (PCAT) Provider Part, representing six primary care domains: ongoing care, coordination (i.e., referrals and information systems), comprehensiveness (i.e., service available and service provided), family-centeredness, community orientation and cultural competence.

**Results:**

Of 401 participating physicians, 163 (40.6 %) were GPs. The total PCAT score was 26.32 ± 2.24 which was the sum score of the six domains and represent the quality of primary care. GPs achieved significantly different total scores and scores on three individual scales: comprehensiveness: service available, comprehensiveness: service provided and community orientation. Multiple linear regressions revealed GPs had a higher total score and scores for comprehensiveness: service provided and community orientation after adjusting for sociodemographic characteristics. In addition, GPs were more likely to intend to stay in their current job in the coming year, and this was associated with their educational level.

**Conclusions:**

Our findings showed that GPs reported higher quality of primary care than other physicians, and were more inclined to stay in their current job. With more comprehensive care and community orientation provided by GPs, residents could reach basic medical cares and needn’t to crowd into larger hospitals.

## Background

Primary care plays a vital role in maintaining the health of patients and decreasing health care costs [1–3]. High quality primary care is associated with an increase in health care utilization, excellent compliance with prescribed treatment regimens, few missed appointments and a positive health status of the population [4–6].

China launched medical reforms in 2009, with the general practitioner system considered a priority [7] and the general practitioner at the core of primary health care. China has developed from a market-oriented health system to a three-tier health system [8], with community health service institutions at the bottom, secondary hospitals in the middle, and tertiary hospitals at the top. Primary care can be provided by general practitioners (GPs) and other physicians (non-GPs). Most of the GPs in China are based in “grass-root” settings [9], that is, urban community health centers (urban CHCs), community health stations (branches of urban CHCs), township health centers (rural CHCs), village clinics and other primary care facilities, while most non-GPs are based in secondary and tertiary hospitals.

However, the utilization of community health service institutions in China is relatively low, suggesting a number of problems in the GP system. These include the weak gate-keeping role of GPs, a lack of effective referral system between the three tiers of health service institution [8], and a lack of trust in GPs by the public [10]. The latter point is especially true–Chinese patients tend to consider the quality of primary care provided by GPs as being much lower than that of non-GPs and usually choose to attend large hospitals directly [11]. As a consequence, issues such as overcrowding in the larger hospitals, rapidly rising health care costs which may lead to inequity in the process of treatment, so the health service being perceived as “too difficult to access and too expensive,” are becoming more and more significant. Many GPs leave their positions [12], seeking better personal development in larger hospitals, making the already weak GP system weaker. We need to know if the quality of primary care offered by Chinese GPs is really lower than that of other physicians and what factors influence the intention of Chinese GPs to stay on in their current job. However, to our knowledge, there is very little previous research on this subject.

Although a number of previous studies concentrate on the quality of primary care provided by GPs and non-GPs, they have limitations. First, research has been mainly concerned with the quality of primary care for sub-populations and specific diseases, for example, migrants, children, older people, and chronic disease [6, 13–15]. Second, with the qualitative approaches applied by some researchers [15, 16], the operational process is complex and results cannot be quantified or generalized. Third, limited sample sizes and low response rates (e.g., 80 % [17]) inevitably flaw results and possibly weaken confidence in the conclusions drawn. Finally, due to insufficient evidence, studies have not reached a reliable conclusion about the quality of primary care provided by GPs and emergency physicians [18]. In view of these shortcomings, quantitative research focusing on the whole population and non-specific diseases, with a relatively large sample size and response rate, is urgently needed.

We seek to overcome the prejudice of Chinese residents against GPs and rebuild concepts in the healthcare-seeking behavior of people. Applying an objective assessment tool, this study aimed to compare the quality of primary care offered by GPs and non-GPs, as well as explore the independent predictors of the future work intentions of GPs, providing information to assist in establishing and perfecting the GP system in China and other developing countries.

## Methods

### Study design and participants

A cross-sectional observational survey was conducted in Guangdong province, China. The subjects were doctors offering primary care in Guangdong province. For the purpose of this study, a GP is defined as a doctor whose scope of practice is the specialty of general medicine. With 21 prefecture-level cities, Guangdong province, the most populous province in China, has more than 105 million people, accounting for 7.82 % of the national population [19]. With 46,534 medical institutions of various kinds, Guangdong province has the second largest number of doctors in the country and a doctor-to-population ratio of 15.05 per 10,000.

We assumed a significance level *∂* = 0.05 and relative error of *δ* = 0.10, providing an ideal sample size of 385 according to *Z*_∂/2_^2^/*δ*^2^.

Researchers from the School of Public Health of Sun Yat-sen University in Guangdong, China, conducted the primary data collection. Informed consent was obtained from all participating study subjects. The Institutional Review Board of Sun Yat-sen University reviewed and approved the protocol of the study in compliance with the Declaration of Helsinki – Ethical Principles for Medical Research Involving Human Subjects (No. IRB2014.9).

### Data collection

Multi-stage random cluster sampling was used to make the sample representative. First, all 21 cities in Guangdong province were divided into two layers according to their annual per capita GDP (<$10,000 USD; ≥$10,000 USD), then two cities were randomly sampled from each layer. The medical institutions in each city were divided into two regional areas (urban and rural). Then three tertiary or secondary hospitals and six urban CHCs were randomly selected from each urban area, and three rural hospitals and six rural CHCs were randomly selected from each rural area. Finally, doctors in internal and traditional Chinese medicine, and pediatric, emergency, obstetric and gynecology departments were randomly sampled from the tertiary and secondary hospitals; while doctors in general consulting rooms, traditional Chinese medicine, and emergency and pediatric departments were randomly selected from the urban and rural CHCs.

The data were collected from November 2013 to September 2014. All 14 survey investigators received appropriate training by leading researchers. The Health Department of Guangdong Province sent investigation letters to the selected facilities to enhance compliance with the survey. Physicians agreeing to participate signed informed consent and completed the questionnaires. A total of 403 doctors were selected, including two doctors who declined to participate. A total of 401 effective questionnaires were collected, consisting of 163 GPs and 238 non-GPs, giving a response rate of 99.5 %.

### Measures

The Primary Care Assessment Tool (PCAT) Provider Version was applied for data collection. The PCAT, consisting of four parts: consumer–client, facility and provider surveys, and a health system survey (in development), was developed by the Johns Hopkins Primary Care Policy Center to measure the quality of primary care services delivered in different settings. The questionnaire, which is widely used and has demonstrated reliability and convergent validity [20], takes about 20 min to complete [12, 21–24]. The instruments were translated into Chinese version under the approval of the Johns Hopkins Primary Care Policy Center. Our pilot field test showed the well-translated questionnaire had excellent reliability (Cronbach’s α = 0.815).

The PCAT section we used consists of eight scales representing six domains: ongoing care, coordination (i.e., referrals and information systems), comprehensiveness (i.e., service available and service provided), family-centeredness, community orientation and cultural competence. Ongoing care refers to the longitudinal use of a regular source of care over time, regardless of the presence or absence of disease or injury. Coordination care is the linking of health care visits and services so that patients receive appropriate care for all of their health problems – physical as well as mental. Comprehensiveness refers to the availability of a wide range of services in primary care and their appropriate provision, across the entire spectrum of types of needs for all but the most uncommon problems in the population, by a primary care provider. Family-centeredness recognizes the family as a major participant in the assessment and treatment of a patient. Community orientation refers to care delivered in the context of the community instead of focus on individual health care. Cultural competence refers to care that honors and respects the beliefs, interpersonal styles, attitudes and behaviors of people as they influence health.

All items in the PCAT were represented by a 4-point Likert-type scale with one indicating “Definitely Not,” two indicating “Probably Not,” three indicating “Probably,” and four indicating “Definitely.” The average score for each scale was derived by averaging the values for all the items under each scale. The average score for overall quality of primary care was derived by averaging the values for all scales. The higher average score, the better performance participants make in the corresponding scale. Missing item or non-response was managed according to the scoring instructions provided with the tool.

The survey also included questions on sociodemographic characteristics (i.e., gender, age, education, working site, working intensity, professional training, income, heath status) and future work intentions.

### Analysis

Mean and standard deviation were applied for the description of average score. The differences in sociodemographic characteristics and quality of primary care measured by PCAT across healthcare provider types (GPs and non-GPs) were tested by bivariate chi square for categorical variables and by *t* test for continuous variables. In order to determine the association between healthcare provider types and quality of primary care, multiple linear regressions were performed after controlling for sociodemographic characteristics of providers. Finally, logistic regression was used to determine the independent predictors of GPs’ intention to stay. All analyses were conducted using SAS 9.2 for Windows.

## Results

### Characteristics of the respondents

As shown in Table 1, the sample consisted of 401 physicians: 163 (40.6 %) were GPs, others (238, 59.4 %) were non-GPs with the majority specialty internal medicine (75/238, 31.5 %) and traditional Chinese medicine (53/238, 22.3 %), other specialties including pediatric (41/238, 17.2 %), emergency (36/238, 15.1 %), obstetric and gynecology (25/238, 10.5 %) and unknown (8/238, 3.4 %). The mean age of respondents was 37.7 years (SD: 8.3 years, range 20–60 years), 59.6 % were male and 66.7 % had bachelor degrees. Of the respondents, 43.9 % worked in rural areas, 47.1 % reported a heavy workload, and the majority (82.3 %) had professional-training opportunities in the last year. The average income was 4667 Chinese Yuan (762 USD); 84.3 % were willing to stay in their current job, and most (88.3 %) were in good health by self-report.

**Table 1:** Demographic, socioeconomic, and health measures of the respondents in Guangdong Province by type of healthcare providers

Variables	Total	GPs	Non-GPs
	*N* = 401(372 ~ 401)	*N* = 163(150 ~ 163)	*N* = 238(220 ~ 238)
	*N *(*%*)	*N *(*%*)	*N *(*%*)
Gender			
Male	239 (59.60)	89 (54.60)	150 (63.03)
Female	162 (40.40)	74 (45.40)	88 (36.97)
Age^*^			
<35	159 (40.56)	59 (37.34)	100 (42.74)
35~	151 (38.52)	73 (46.20)	78 (33.33)
45~	82 (20.92)	26 (16.46)	56 (23.93)
Education^**^			
<Bachelor	130 (33.33)	77 (47.83)	53 (23.14)
≥Bachelor	260 (66.67)	84 (52.17)	176 (76.86)
Working site^**^			
Country hospital	88 (21.95)	11 (6.75)	77 (32.35)
Rural CHC	88 (21.95)	55 (33.74)	33 (13.87)
Tertiary hospital	76 (18.95)	4 (2.45)	72 (38.24)
Secondary hospital	40 (9.98)	4 (2.45)	36 (15.13)
Urban CHC	109 (27.18)	89 (54.60)	20 (8.40)
Working intensity^*^			
Light	202 (52.88)	87 (58.00)	115 (49.57)
Heavy	180 (47.12)	63 (42.00)	117 (50.43)
Prof-training			
No	71 (17.71)	23 (14.11)	48 (20.17)
Yes	330 (82.29)	140 (85.89)	190 (79.83)
Income^**^			
<3000	79 (21.24)	38 (25.00)	41 (18.64)
3000~	149 (40.05)	72 (47.37)	77 (35.00)
5000~	144 (38.71)	42 (27.63)	102 (46.36)
Intent to stay^*^			
No	63 (15.71)	17 (10.43)	46 (19.33)
Yes	338 (84.29)	146 (89.57)	192 (80.67)
Health status			
Not well	47 (11.72)	14 (8.59)	33 (13.87)
Well	354 (88.28)	149 (91.41)	205 (86.13)

^*^ *P* < 0.05; ^**^ *P* < 0.01; based on Chi-square test of difference across healthcare providers

There were a number of significant differences between GPs and non-GPs in terms of these characteristics. GPs were much younger and more likely to have lower level qualifications (below bachelor degree). They mainly worked in CHCs, with relatively lower working intensity and less income. However, GPs were more likely to plan to stay in their current job in the coming year.

### Individual and total primary care attributes scores

The total PCAT scores reported by the participants were high (Table 2). The combined total score of the eight scales was 26.32 ± 2.24. Community orientation scored less than 3 and was lower than that of other scales.

**Table 2:** Individual and total primary care attributes scores reported by respondents by type of healthcare providers

Variables	Total	GPs	Non-GPs
	*N* = 401	*N* = 163	*N* = 238
Ongoing Care	3.10 ± 0.41	3.11 ± 0.42	3.10 ± 0.40
Coordination	3.45 ± 0.40	3.45 ± 0.39	3.46 ± 0.41
Coordination-information systems	3.40 ± 0.48	3.37 ± 0.45	3.42 ± 0.50
Comprehensiveness-service available^*^	3.33 ± 0.55	3.41 ± 0.46	3.27 ± 0.59
Comprehensiveness-service provided^**^	3.18 ± 0.37	3.24 ± 0.37	3.13 ± 0.37
Family-centeredness	3.52 ± 0.43	3.48 ± 0.47	3.54 ± 0.39
Community Orientation^**^	2.90 ± 0.70	3.18 ± 0.55	2.70 ± 0.73
Culturally Competent	3.44 ± 0.49	3.43 ± 0.51	3.44 ± 0.47
Total score^**^	26.32 ± 2.24	26.67 ± 2.19	26.07 ± 2.25

^*^ *P* < 0.05; ^**^ *P* < 0.01; based on *t* test of difference across healthcare providers

Compared with non-GPs, the scores of three individual scales were higher in GPs, meaning GPs performed better in these three fields: comprehensiveness: service available, comprehensiveness: service provided, and community orientation. More importantly, the total score of GPs was significantly higher than that of non-GPs (26.67 ± 2.19 vs. 26.07 ± 2.25, *P* < 0.01), indicating that GPs’ quality of primary care may be considerably better than non-GPs.

### Provider characteristics associated with the quality of primary care

After controlling for sociodemographic characteristics, GPs continued to have a higher total PCAT score, adding strong evidence to suggest that GPs’ quality of primary care was better than non-GPs (Tables 3 and 4). Multiple linear regression showed GPs had higher scores in two individual scales: comprehensiveness: service provided and community orientation.

**Table 3:** Provider characteristics associated with individual and total primary care attributes using multiple linear regression

Variables	Ongoing Care	Coordination	Coordination- information systems	Comprehensiveness-service available	Comprehensiveness-service provided
	β (SE)	β (SE)	β (SE)	β (SE)	β (SE)
Intercept	2.93 (0.10)	3.21 (0.09)	3.17 (0.11)	3.08 (0.13)	3.03 (0.09)
GP(ref: Non-GP)					
GP	0.02 (0.04)	−0.02 (0.04)	−0.08 (0.05)	0.09 (0.06)	0.1^* ^(0.04)
Gender					
(ref: Female)					
Male	0.01 (0.04)	−0.06 (0.04)	−0.04 (0.05)	−0.05 (0.06)	−0.02 (0.04)
Age(ref: <35)					
35~	0.04 (0.05)	0.07 (0.05)	0.10 (0.06)	0.05 (0.07)	0.09^*^ (0.04)
45~	0.02 (0.06)	0.04 (0.06)	0.08 (0.07)	−0.01 (0.08)	0.11^* ^(0.05)
Education					
(ref: <Bachelor)					
≥Bachelor	0.04 (0.05)	−0.0004 (0.05)	0.05 (0.06)	−0.04 (0.07)	0.04 (0.05)
Working intensity					
(ref: Light)					
Heavy	0.05 (0.04)	0.04^*^ (0.04)	−0.02 (0.05)	0.01 (0.06)	−0.0003 (0.04)
Prof-training					
(ref: No)					
Yes	0.05 (0.06)	0.12^*^ (0.05)	0.21^**^ (0.07)	0.25^**^ (0.08)	0.08 (0.05)
Income(ref: <3000)					
3000~	−0.06 (0.06)	−0.04 (0.06)	−0.12 (0.07)	−0.04 (0.08)	−0.09 (0.05)
5000~	−0.09 (0.06)	−0.05 (0.06)	−0.12 (0.08)	−0.08 (0.09)	−0.11^*^ (0.06)
Health status					
(ref: Not well)					
Well	0.13^* ^(0.06)	0.18^** ^(0.06)	0.15^*^ (0.07)	0.10 (0.09)	0.04 (0.06)
Adjusted *R* ^*2*^	0.0034	0.057	0.0297	0.0243	0.0494
*N*	401	401	401	401	401

^*^ *P* < 0.05; ^**^ *P* < 0.01

**Table 4:** Provider characteristics associated with individual and total primary care attributes (cont.)

Variables	Family-centeredness	Community Orientation	Culturally Competent	Total score
	β (SE)	β (SE)	β (SE)	β (SE)
Intercept	3.26 (0.10)	2.18 (0.15)	3.14 (0.11)	24.01 (0.51)
GP (ref: Non-GP)				
GP	−0.05 (0.05)	0.40^**^ (0.07)	0.02 (0.05)	0.48^*^ (0.24)
Gender (ref: Male)				
Female	0.05 (0.04)	0.13^*^ (0.07)	0.05 (0.05)	0.08 (0.22)
Age (ref: <35)				
35~	0.03 (0.05)	0.18^*^ (0.08)	−0.002 (0.06)	0.56^*^ (0.26)
45~	−0.03 (0.06)	0.26^**^ (0.09)	0.07 (0.07)	0.54 (0.31)
Education (ref: <Bachelor)				
≥Bachelor	0.03 (0.05)	−0.001 (0.08)	0.01 (0.06)	0.12 (0.27)
Working intensity				
(ref: Light)				
Heavy	0.03 (0.04)	−0.04 (0.07)	0.02 (0.05)	0.10 (0.22)
Prof-training (ref: No)				
Yes	0.14^*^ (0.06)	0.37 (0.09)	0.15^*^ (0.07)	1.37 (0.30)
Income (ref: <3000)				
3000~	−0.03 (0.06)	−0.14 (0.09)	−0.07 (0.07)	−0.58 (0.31)
5000~	0.01 (0.07)	−0.36^**^ (0.10)	−0.003 (0.08)	−0.80^*^ (0.34)
Health status (ref: Not well)				
Well	0.12 (0.07)	0.28^**^ (0.10)	0.18^*^ (0.08)	1.18^**^ (0.34)
Adjusted *R* ^*2*^	0.0206	0.1886	0.0493	0.1005
*N*	401	401	401	401

^*^ *P* < 0.05; ^**^ *P* < 0.01

The covariates of age, income, and health status were also significantly associated with the overall quality of primary care. Specifically, physicians between the ages of 35 and 45 years, with incomes lower than 3000 Yuan and with better health status reported higher overall scores.

### Predictors of providers’ intention to stay

After controlling for some covariates GPs were more likely, compared to non-GPs, to intend staying in their current job in the next year (OR: 2.34, 95 % CI: 1.17–4.7). Physicians with higher educational levels were also more inclined to stay in their present job (Table 5).

**Table 5:** Factors associated with providers’ intent to stay through logistic regression

Model (*N* = 401)	*OR* (95 % *CI*)
GP	
No	1.00
Yes	2.34^*^ (1.17–4.7)
Gender	
Female	1.00
Male	0.55 (0.29–1.04)
Age	
<35	1.00
35~	0.77 (0.38–1.54)
45~	1.28 (0.54–3.03)
Education	
<Bachelor	1.00
≥Bachelor	2.11^*^ (1.01–4.42)
Working intensity	
Light	1.00
Heavy	1.00 (0.54–1.84)
Prof-training	
No	1.00
Yes	1.18 (0.57–2.46)
Income	
<3000	1.00
3000~	0.91 (0.37–2.25)
5000~	0.55 (0.21–1.42)
Health status	
Not well	1.00
Well	0.99 (0.4–2.47)
Adjusted *R* ^2^	0.1668

^*^ *P* < 0.05

## Discussion

This is one of few studies that have investigated the quality of primary care provided by GPs and other physicians in China. Our key findings included: 1) the quality of primary care provided by GPs was reportedly better than that of non-GPs, particularly for comprehensiveness: service provided and community orientation; and 2) GPs were more likely to stay in their current job than non-GPs, after controlling for sociodemographic characteristics.

The use of research tools to assess the quality of primary care may assist in the improvement of services and PCAT is the most adequate tool for this purpose [25]. The scores of PCAT obtained in our study were comparable to studies in other countries. A study from Brazil found that the Community Health Service had a significantly higher overall PCAT score compared to other types of public primary healthcare providers [26]; this is comparable with our results, which showed that 88.34 % of GPs worked in the community and provided better quality of primary care. A considerable amount of research using the PCAT: Adult Edition has taken place, with the majority of patients reporting better primary care experiences from GPs than non-GPs. A study from South Korea showed that primary care physicians (i.e., family physicians and general practitioners) could provide superior performance compared to non-primary care physicians [27]. This suggests that GPs provide better quality of primary care than non-GPs, from both provider and patient perspectives, although this general trend might be partly different among countries with large differences.

**Fig. 1:**
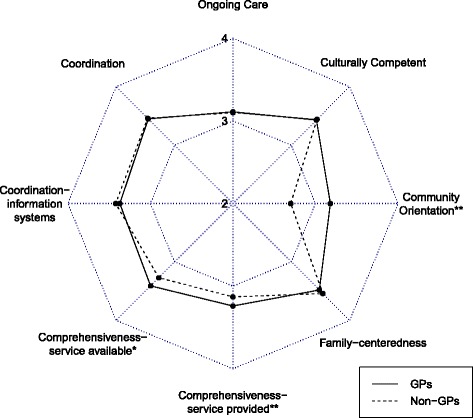
Primary care attributes: general practitioners versus other physicians

Equitable primary care is yet to be strengthened with regard to the community orientation attribute (Fig. 1) and Wang et al. had similar findings from a patient perspective [28]. Community-oriented primary care (COPC) is the combination of traditional public health and clinical medicine. It has proven to be beneficial in identifying local health issues and in using limited health resources to meet the majority needs of the community [29]. Therefore, a balanced professional force of general practitioners should be developed by introducing international standards of medical education and medical training [30].

Our study found that GPs were more inclined to stay in their present job than non-GPs. It is viewed as a positive outcome for Chinese government efforts that GPs, by providing first-contact care for the population, play the role of gatekeeper to the healthcare system, even though this first-contact system has not yet been completely established throughout China [31]. Like many other developing countries, China has, in recent years, realized the importance of GPs and made major efforts in their education, training, and career development [32]. There are two main approaches for training GPs in China. One is “5 + 3” model, that is, a student first received 5 years of clinical medicine (including traditional Chinese medicine) undergraduate education then 3 years of GP’ standardized training. The other is rotating station training, that is, the primary care practitioners and assistant practitioners who meet the requirements are trained for 1 to 2 years respectively. The government also take some measures to broaden the career development path of GPs such as, improving their wages and working environment, reinforcing their skill training and medical practice levels, giving the policy tilt towards their promotion.

Several limitations should be noted when interpreting our findings. First, the data was based on self-reporting by physicians, assessing solely their perceptions of health care experiences but ignoring aspects of the technical quality of medical care. Second, the study was a cross-sectional design, so causal inferences could not be established. Third, this study was conducted in one province with a limited sample size, so the generalizability of the findings nationally is limited.

Despite these limitations, our findings provide guidance to government in formulating health care policy, and have potential to change Chinese patients’ concept of medical treatment and guide their healthcare-seeking behavior. Although specialists in secondary or tertiary hospitals appear better equipped, the total quality of primary care provided is inferior to that of GPs. Policymakers must address a critical need for GPs, in order to improve quality of primary care, and vigorously promote and guide residents’ proper healthcare-seeking behavior.

## Conclusions

We found the quality of primary care reported by GPs was better than that reported by non-GPs, particularly in comprehensiveness: service provided and community orientation. We also found GPs were more likely to stay in their current job than non-GPs. This implies that the government trying to change the concept of medical treatment of Chinese patients and guide their healthcare-seeking behavior could have a great effect on the improvement of medical climate.
